# Effect of far-infrared therapy device on arteriovenous fistula maturation and lifespan in hemodialysis patients: a randomized controlled clinical trial

**DOI:** 10.3389/fsurg.2023.1260979

**Published:** 2023-09-11

**Authors:** Jianqiang Huang, Peilan Zheng, Xiaobin Chen, Fan Zheng, Beibei He

**Affiliations:** ^1^ Department of General Surgery, No. 900th Hospital of China People's Liberation Army Joint Logistics Support Force, Fuzhou, China; ^2^ Department of Nephrology, No. 900th Hospital of China People's Liberation Army Joint Logistics Support Force, Fuzhou, China

**Keywords:** far infrared therapy, mucopolysaccharide polysulfate cream, arteriovenous fistula, therapeutic effect, patency rate

## Abstract

**Introduction:**

Arteriovenous fistula (AVF) is the first choice of vascular access for hemodialysis treatment, and its surgical maturity rate is not high, and its postoperative complications (mostly stenosis) significantly shorten its life. At present, there are few studies on treatment methods to improve the maturity and survival of AVF. In this study, the effect of far infrared therapy (FIR) on the maturity and longevity of arteriovenous fistula in hemodialysis patients was discussed, and the protective mechanism of AVF induced by FIR therapy was explored, aiming at exploring a new treatment method.

**Methods:**

The hemodialysis patients admitted to the 900th Hospital of the Chinese Joint Logistics Support Force of the People's Liberation Army from January 2021 to April 2023 were randomly divided into control group and intervention group, with 40 cases in each group. Among them, the control group was coated with mucopolysaccharide polysulfonate cream; Intervention group: The patients were treated with mucopolysaccharide polysulfonate cream and far infrared radiation at the same time. After 3 months' intervention, the arteriovenous fistula (vein diameter, mature time of arteriovenous fistula, blood flow controlled by pump during dialysis, blood flow of brachial artery during dialysis and the occurrence of complications of internal fistula (oozing, occlusion and infection) and the pain score (numerical rating scale, NRS) of the two groups were compared, and the curative effects were compared.

**Results:**

There was no significant difference in general data between the two groups (*P* > 0.05), which indicated that the study was comparable. After 3 months' intervention, the vein diameter, pump-controlled blood flow and brachial artery blood flow in the intervention group were significantly higher than those in the control group (*P* < 0.05). And the maturity time, NRS score and complication rate of arteriovenous fistula were significantly lower than those of the control group (*P* < 0.05). The primary patency rate of AVF in the intervention group was higher than that in the control group, and the overall patency rate between the two groups was statistically significant (*P* < 0.05).

**Conclusions:**

As a promising new treatment method, far infrared therapy can effectively promote the maturity of AVF, increase venous diameter, pump controlled blood flow during dialysis, brachial artery blood flow during dialysis, and prolong the service life of AVF.

## 1. Introduction

In recent years, the number of patients with end-stage renal disease who need hemodialysis treatment in the world is increasing year by year (1). In order to get the best dialysis treatment, patients often rely on a stable and effective hemodialysis pathway (2). At present, there are three ways of hemodialysis access, namely, autogenous arteriovenous fistula (AVF), arteriovenous graft (AVG) and central venous catheter (CVC), among which AVF is the preferred vascular access (80%–85%) with lower complications and better primary patency rate than the other two ways (3). In a multivariate analysis, Bray et al. (4) found that compared with AVF patients, the death risk of tunnel CVC patients increased by 7 times (mostly due to sepsis). As the first choice of vascular access, AVF is influenced by many factors, which limits its long-term application in hemodialysis treatment. On the one hand, uremic patients are often complicated with risk factors (such as hypertension and diabetes) that affect the function of internal fistula (5); On the other hand, with the increasing use of internal fistula, repeated puncture during hemodialysis can easily lead to infection, vascular endothelial injury, stenosis and thrombosis, thus affecting the service life of AVF (6, 7). According to reports, the patency rates of AVF in one year and two years are 50%–70% and 30%–40% respectively (8). Therefore, it is very important for uremia patients to screen and prevent AVF-related complications and maintain the function of internal fistula to ensure the smooth progress of hemodialysis.

At present, drugs (mucopolysaccharide polysulfate cream, aspirin) and other routine treatments are mainly used in clinic to effectively prolong the life of AVF and ensure the smooth hemodialysis treatment of uremia patients (9). As a new treatment method, far infrared ray therapy has been recently applied to the treatment of internal fistula and achieved good results (10). It can improve the blood flow of AVF in hemodialysis patients to some extent by infrared radiation, thus improving the dialysis quality of patients (11). However, at present, there are few reports on this aspect. This study analyzes the curative effect of far-infrared therapeutic instrument combined with mucopolysaccharide polysulfonate cream on internal fistula function of hemodialysis patients by collecting patients with end-stage renal failure in our hospital, aiming at serving the clinic.

## 2. Methods

### 2.1. Data collection

The clinical data of hemodialysis patients admitted to the 900th Hospital of the Chinese Joint Logistics Support Force of the People's Liberation Army from January 2021 to April 2023 were selected. Inclusion criteria: ① Chronic renal failure was diagnosed, and met the indication of hemodialysis treatment, that is, serum creatinine >707 µmol/L; ② Age ≥18 years; ③ All patients underwent forearm arteriovenous fistula plasty for the first time; ④ Agree to participate in the study and sign the informed consent form. Exclusion criteria: ① patients with heart, liver and other organ failure; ② Patients with coagulation dysfunction; ③ Patients with consciousness disorder or mental illness who can't cooperate with the study; (4) those with incomplete information.

### 2.2. Therapeutic method

In order to ensure the authenticity and reliability of the research results, the subjects included in this study were screened strictly according to the inclusion and exclusion criteria, and the subjects were randomly divided into control group and intervention group by random number table method, with 40 cases in each group. Patients in both groups were treated with routine measures to protect arteriovenous fistula, including proficient operational techniques (correct puncture and compression to stop bleeding, and appropriate pump speed during hemodialysis). Control group: One day after each dialysis, hot towel was used to compress the limbs of AVF for 15 min (the temperature was controlled at 45°C), then appropriate amount of mucopolysaccharide polysulfonate cream was evenly applied around the limbs of AVF, and massage was performed for 15 min to promote the absorption of the ointment. Intervention group: In addition to the treatment in the control group, far infrared ray therapy was combined, that is, far infrared ray therapeutic instrument was used to irradiate limbs of arteriovenous fistula for 30 min before each hemodialysis (the instrument probe should be about 20 cm away from AVF and the temperature should be less than 40°C during the operation). Patients in both groups were treated on non-dialysis days, and the treatment cycle was up to 3 months. The subjective data of patients before and after treatment were statistically analyzed by two medical staff with rich professional knowledge.

### 2.3. Indicators collection

General information such as sex, age, primary disease, duration of hemodialysis, location of arteriovenous fistula; Before and after treatment, the internal diameter of vein, the maturity time of arteriovenous fistula, the pump-controlled blood flow during dialysis and the blood flow of brachial artery during dialysis; Incidence of AVF-related complications (oozing, occlusion, infection) after treatment; Pain score of patients before and after treatment [numerical rating scale (NRS)] (12), etc. The clinical efficacy of the control group and the intervention group were compared and evaluated. The set score of NRS is 0–10, which is divided into three grades: light, medium and heavy, of which mild pain is 0–3; Moderate pain: 4–6 points; Severe pain: 7–10 points. The NKF-K/DOQI glossary defines fistula maturation as: blood flow >600 ml/min, a diameter >6 mm, and a depth of within 6 mm (13).

### 2.4. Follow-up

All postoperative patients were followed up regularly, and the follow-up period was once every 3–6 months, and the follow-up was conducted by outpatient service, SMS, telephone and WeChat. Follow-up included the patient's demographic indicators, clinical biochemical indicators and the long-term use of AVF. The deadline for follow-up is July 6, 2023 or AVF failure.

### 2.5. Statistical analysis

SPSS 26.0 software and R(4.0.4) software were used for statistical analysis of the data. The measurement data of normal distribution were expressed as (*x* ± *s*), and *t*-test was used for comparison between groups. The counting data were expressed by *n*(%), and the comparison between groups was conducted by *χ*^2^ test or Fisher exact test (*n* < 5). Spearman correlation analysis was used for correlation analysis; Kaplan-Meier method was used to draw the survival curve, and the primary patency rate of AVF in two groups was analyzed. Log-rank was used to compare the difference of survival time between the two groups. *P* < 0.05 was statistically significant.

## 3. Results

### 3.1. General data

A total of 80 patients were included in this study, including 43 males (53.75%) and 37 females (46.25%), aged from 26.0–69.0 (51.38 ± 7.18). According to the random number table, 80 patients were divided into control group and intervention group with 40 cases in each group. There was no significant difference in general data characteristics between the two groups (*P* > 0.05), which indicated that they were clinically comparable, as shown in Table 1.

**Table 1:** Comparison of the characteristics of two groups of general data.

Variable	Control group	Intervention group	*t*/*χ*^2^	*P*
Age (years)	51.48 ± 5.95	51.28 ± 8.30	−0.124	0.902
Gender			0.050	0.823
Male	22 (55.00%)	21 (52.50%)		
Female	18 (45.00%)	19 (47.50%)		
Primary disease			0.933	0.920
Hypertensive nephropathy	12 (30.00%)	10 (25.00%)		
Diabetic nephropathy	11 (27.50%)	11 (27.50%)		
Chronic glomerulonephritis	9 (22.50%)	8 (20.00%)		
Polycystic kidney	5 (12.50%)	8 (20.00%)		
Others	3 (7.50%)	3 (7.50%)		
Hemodialysis duration (h)	3.28 ± 0.53	3.25 ± 0.47	0.224	0.824
Location			0.287	0.592
Left upper limb	30 (75.00%)	32 (80.00%)		
Right upper limb	10 (25.00%)	8 (20.00%)		

### 3.2. Comparison of pump-controlled blood flow, brachial artery blood flow and maturity time of arteriovenous fistula between the two groups after 3 months of intervention

After 3 months of treatment, the pump-controlled blood flow and brachial artery blood flow in the intervention group were higher than those in the control group, and the difference was statistically significant (*P* < 0.05). However, the maturity time of arteriovenous fistula in the intervention group was significantly shorter than that in the control group, with statistical significance (*P* < 0.05), as shown in Table 2.

**Table 2:** Comparison of pump-controlled blood flow, brachial artery blood flow during dialysis and maturity time of arteriovenous fistula between the two groups after intervention.

Variable	Pump control blood flow during dialysis (ml/min)	Brachial artery blood flow during dialysis (ml/min)	Maturity time of arteriovenous fistula (d)
Control group	212.50 ± 17.72	614.11 ± 44.06	42.45 ± 4.36
Intervention group	253.75 ± 29.35	701.00 ± 49.32	31.85 ± 5.43
*t*	−7.610	−9.170	9.628
*P*	<0.001	<0.001	<0.001

### 3.3. Comparison of vein diameter and NRS score between the two groups before and after intervention

Comparing the differences of NRS score and vein diameter between the two groups before intervention, the results showed that there was no significant difference in NRS score and vein diameter between the control group and the intervention group (*P* > 0.05). After 3 months of treatment, the NRS score in the intervention group was significantly lower than that in the control group (*P* < 0.05), while the internal diameter of vein was significantly higher than that in the control group (*P* < 0.05). In addition, after treatment, the NRS scores of the control group and the intervention group were lower than those before intervention (*P* < 0.05), while the vein diameters of the control group and the intervention group were higher than those before intervention (*P* < 0.05), as shown in Table 3.

**Table 3:** Comparison of NRS score and vein diameter between the two groups before and after intervention for 3 months.

Variable	NRS score		Venous diameter	
	Before treatment	After treatment	Before treatment	After treatment
Control group	6.45 ± 1.15	5.70 ± 1.00*	3.56 ± 0.19	5.12 ± 0.32*
Intervention group	6.30 ± 1.02	3.63 ± 0.87*	3.55 ± 0.18	6.15 ± 0.24*
*t*	0.617	9.955	0.117	−16.602
*P*	0.539	<0.001	0.907	<0.001

*The difference before and after intervention is statistically significant, that is, *P* < 0.05.

### 3.4. Comparison of incidence of AVF-related complications

All patients were followed up. In the control group, 22 cases (55.00%) had complications, including 8 cases of Arterial-venous fistulae thrombosis (20.00%), 2 cases of superficial phlebitis (5.00%), 5 cases of subcutaneous hematoma (12.50%) and 7 cases of local bleeding (17.50%). In the intervention group, 12 cases had complications, accounting for 30.00%, including 2 cases of Arterial-venous fistulae thrombosis (5.00%), 3 cases of superficial phlebitis (7.50%), 3 cases of subcutaneous hematoma (7.50%) and 4 cases of local bleeding (10.00%). It shows that the incidence of AVF-related complications in the intervention group is lower than that in the control group, and the difference is statistically significant (*P* < 0.05), as shown in Table 4.

**Table 4:** Postoperative complications of two groups of patients.

Variable	Cases	Arterial-venous fistulae thrombosis	Superficial phlebitis	Ecchymoma	Local bleeding	Total
Control group	40	8	2	5	7	22
Intervention group	40	2	3	3	4	12
*t*/χ^2^	–	4.114	0.213	0.556	0.949	5.115
*P*	–	0.043	0.644	0.456	0.330	0.024

### 3.5. Comparison of primary patency rate of AVF between two groups of hemodialysis patients

**Figure 1:**
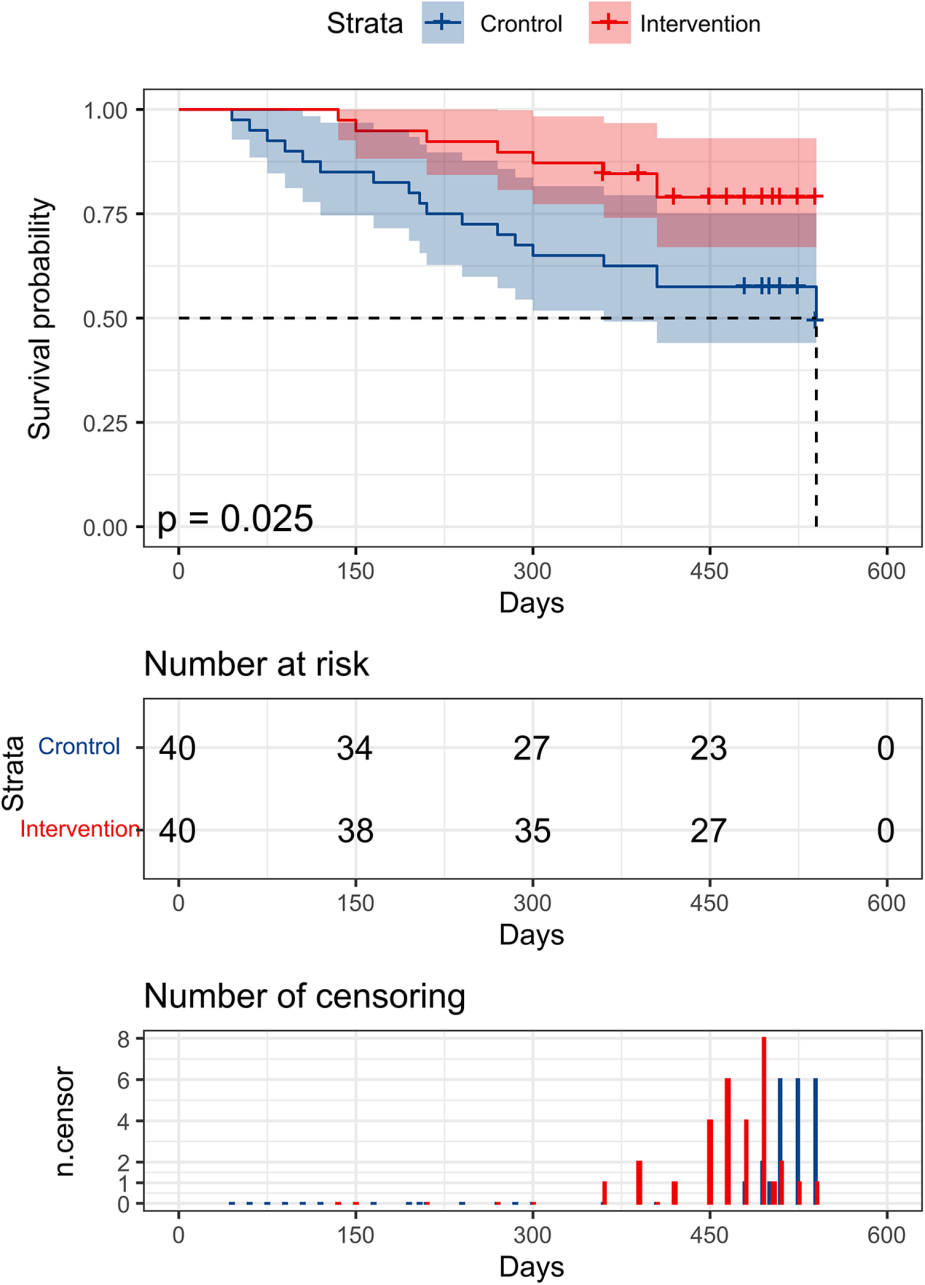
Comparison of survival curves of primary patency rate of AVF between two groups.

Spearmen correlation analysis showed that far infrared therapy was positively correlated with the mature time of AVF fistula (*r* = 0.737, *P* < 0.001). The median follow-up time was 16.5 months, and the average time for AVF to mature was 37.15 ± 7.24 days. In the control group, the primary patency rates of AVF at 12 months and 18 months were 62.5% and 49.3%, respectively. In the intervention group, the primary patency rate of AVF at 12 months and 18 months was 85.0% and 79.5%, respectively. The primary patency rate of patients in the intervention group was higher than that in the control group, and the overall patency rate between the two groups was statistically significant (*χ*^2 ^= 5.313, *P* = 0.025), as shown in Figure 1.

## 4. Discussion

Arteriovenous fistula (AVF), as the first choice for hemodialysis, is the lifeline of patients with chronic renal failure. However, long-term hemodialysis patients have different degrees of vascular sclerosis, and repeated puncture during hemodialysis can easily lead to complications such as internal fistula thrombosis, internal fistula stenosis, pseudoaneurysm formation, phlebitis, etc. (among which internal fistula thrombosis is the most common), which often leads to the decrease of internal fistula blood flow, which is not enough to meet the daily hemodialysis requirements, and in severe cases, it can lead to AVF failure, bringing huge economic pressure to patients’ families and society (14–16). Therefore, it is very important for clinical medical staff to explore and formulate corresponding programs to improve the function of arteriovenous fistula (increase blood flow and reduce related complications) (17). At present, the conventional nursing method (hot compress) is too simple, and the prognosis of some uremic patients has not improved significantly. With the continuous development of medical technology, far-infrared therapeutic apparatus has been applied to the treatment of diseases, and related studies have reported that far-infrared irradiation can reduce inflammatory reaction and increase blood flow, thus improving the maturity rate of AVF (18). In addition, studies have shown that mucopolysaccharide polysulfonate cream is widely used in the treatment of arteriovenous fistula because of its functions of improving blood circulation and protecting vascular endothelium (10, 11). By comparing the differences of NRS score and vein diameter before and after intervention, it was found that the NRS scores of the control group and the intervention group were lower than those before intervention, while the vein diameter was higher than that before intervention (*P* < 0.05). The results showed that both mucopolysaccharide polysulfonate cream and far infrared therapy could effectively improve the function of arteriovenous, which was consistent with previous research results. However, at present, there are relatively few related studies on the combination of the two in the care of arteriovenous fistula. Based on this, this study analyzes the curative effect of far-infrared therapeutic instrument combined with mucopolysaccharide polysulfonate cream on the internal fistula function of hemodialysis patients by collecting patients with end-stage renal failure in our hospital.

The results of this study showed that after 3 months of treatment, the pump-controlled blood flow, brachial artery blood flow and vein diameter in the intervention group were higher than those in the control group (*P* < 0.05), while the maturity time and NRS score of arteriovenous fistula were significantly shorter than those in the control group (*P* < 0.05). It is suggested that far infrared therapy can improve the function of arteriovenous fistula by increasing blood flow, vein diameter and shortening the maturity time of internal fistula. On the one hand, far infrared radiation can deliver heat to the skin to promote blood circulation of local tissues, and it can also improve blood flow by dilating blood vessels, thus improving hemodialysis efficiency (10). Xu Jing (19) also pointed out that far infrared radiation therapy can significantly improve blood flow of AVF through analysis; On the other hand, far infrared irradiation can alleviate the vascular wall hyperplasia caused by inflammatory reaction by reducing the inflammatory reaction, thus improving the internal diameter of AVF blood vessels (20), and further improving the success rate of surgery to a certain extent; Related research has pointed out that the factors affecting the maturity of internal fistula mainly include endothelial injury, insufficient blood flow and vascular stenosis, while far infrared radiation can weaken the oxidative stress of the body and induce anti-inflammation through non-thermal effects, thus reducing intimal hyperplasia, increasing blood flow and finally promoting the maturity of internal fistula (21). Finally, we found that far infrared ray therapy can effectively reduce the NRS score of patients, and consider that far infrared ray can reduce the pain of puncture by reducing the excitability of patients' sensory nerves, and in addition, far infrared ray can improve the phagocytosis of white blood cells through resonance, thus playing an anti-inflammatory role (22).

In this study, patients in the intervention group and the control group were followed up regularly, and the follow-up content was to observe the occurrence of related complications after internal fistula operation and the first-stage patency rate. We found that the incidence of AVF-related complications in the intervention group was lower than that in the control group, and the difference was statistically significant (*P* < 0.05), especially in internal fistula embolism (*P* < 0.05). Considering that far infrared radiation can improve local blood circulation and inhibit platelet aggregation to prevent thrombosis, the incidence of internal fistula embolism can be greatly reduced (23). Finally, through Spearmen correlation analysis, we found that far infrared therapy was positively correlated with the mature time of AVF internal fistula (*r* = 0.737, *P* < 0.001). By comparing the patency rate of arteriovenous fistula in the first stage between the two groups, it was shown that the patency rate of AVF in the first stage in the intervention group was higher than that in the control group (85.0% vs. 62.5%, *P* < 0.05). The study with Lin et al. (24) also pointed out that far infrared therapy can effectively prolong the primary patency rate of AVF, which is consistent with the results of this study.

This study has some limitations. On the one hand, the sample size included in this study is small, and even if the research objects are screened according to the inclusion and exclusion criteria, there is still some selection bias, because this study is not double-blinded; On the other hand, the follow-up time of this study is 18 months, which is relatively short; Therefore, it is necessary to expand the sample size and extend the follow-up time in the later stage to further verify the curative effect of far infrared ray in the treatment of arteriovenous fistula, so as to guide clinical medical staff to formulate diagnosis and treatment plans.

In a word, far infrared therapy can effectively promote the maturity of AVF, increase the internal diameter of vein, control blood flow during dialysis, brachial artery blood flow during dialysis and prolong the service life of AVF, which is expected to become a promising new diagnosis and treatment method of AVF.

## Data Availability

The original contributions presented in the study are included in the article/Supplementary Material, further inquiries can be directed to the corresponding author.
